# Structure and interactions of the *Bacillus subtilis* sporulation inhibitor of DNA replication, SirA, with domain I of DnaA

**DOI:** 10.1111/mmi.12713

**Published:** 2014-08-05

**Authors:** Katie H Jameson, Nadia Rostami, Mark J Fogg, Johan P Turkenburg, Anne Grahl, Heath Murray, Anthony J Wilkinson

**Affiliations:** 1 Structural Biology Laboratory, Department of Chemistry, University of YorkYork, YO10 5DD, UK; 2 Centre for Bacterial Cell Biology, Institute for Cell & Molecular Biosciences, Newcastle UniversityNewcastle upon Tyne, NE2 4AX, UK

## Abstract

Chromosome copy number in cells is controlled so that the frequency of initiation of DNA replication matches that of cell division. In bacteria, this is achieved through regulation of the interaction between the initiator protein DnaA and specific DNA elements arrayed at the origin of replication. DnaA assembles at the origin and promotes DNA unwinding and the assembly of a replication initiation complex. SirA is a DnaA-interacting protein that inhibits initiation of replication in diploid *Bacillus subtilis* cells committed to the developmental pathway leading to formation of a dormant spore. Here we present the crystal structure of SirA in complex with the N-terminal domain of DnaA revealing a heterodimeric complex. The interacting surfaces of both proteins are α-helical with predominantly apolar side-chains packing in a hydrophobic interface. Site-directed mutagenesis experiments confirm the importance of this interface for the interaction of the two proteins *in vitro* and *in vivo*. Localization of GFP–SirA indicates that the protein accumulates at the replisome in sporulating cells, likely through a direct interaction with DnaA. The SirA interacting surface of DnaA corresponds closely to the HobA-interacting surface of DnaA from *Helicobacter pylori* even though HobA is an activator of DnaA and SirA is an inhibitor.

## Introduction

Across the kingdoms of life, DNA replication is tightly regulated to ensure co-ordination with cell growth and development. Failure to maintain and control chromosome copy number is frequently associated with disease or cell death. Regulation of DNA replication is mainly exerted at the initiation step when an initiator protein binds to the origin of replication and promotes the assembly of a nucleoprotein complex from which replication forks diverge.

In prokaryotes, the DNA replication initiator protein is DnaA. In its ATP-bound state, DnaA assembles at the origin of replication, *oriC*, by binding to a number of 9 bp recognition sequences termed DnaA-boxes (Yoshikawa and Ogasawara, 1991). Recruitment of DnaA to *oriC* is believed to generate a helical oligomer of DNA-bound DnaA that promotes duplex unwinding at an AT-rich region within the origin termed the DNA unwinding element (Duderstadt *et al.*, 2011). DnaA is a member of the AAA+ (ATPases associated with diverse cellular activities) protein superfamily and is made up of four distinct domains (Kaguni, 2006). Domain I is known to have a number of interaction partners, including replication regulators and DNA helicase (Seitz *et al.*, 2000; Abe *et al.*, 2007). Domain II is thought to be a flexible linker, that may allow nuances in regulatory control (Molt *et al.*, 2009). Domain III binds and hydrolyses ATP, mediates DnaA oligomerization (Erzberger *et al.*, 2006) and binds single stranded DNA thus aiding duplex unwinding (Duderstadt *et al.*, 2011). Domain IV binds double stranded DNA, interacting with the DnaA-box motifs (Fujikawa *et al.*, 2003). The binding of a threshold level of DnaA–ATP at *oriC* leads to duplex unwinding and the recruitment of other initiation proteins (Leonard and Grimwade, 2011).

Although DnaA is conserved in bacteria, many of the other initiation components are not. This is exemplified by differences in replication initiation observed between *E. coli* and *B. subtilis*, organisms which provide our most thorough understanding of the control of replication initiation in Gram-negative and Gram-positive bacteria, respectively. An important early step following duplex unwinding in both organisms, is the recruitment of a DNA helicase (*Ec* DnaB/*Bsu* DnaC) to the origin where it is loaded onto the DNA by a helicase loader (*Ec* DnaC/*Bsu* DnaI). This is followed by the binding of the primase, DnaG. Curiously, replication initiation in *B. subtilis* requires two additional proteins, DnaD and DnaB, neither of which is present in *E. coli* (Ishigo-oka *et al.*, 2001; Rokop *et al.*, 2004) – it should be noted that *B. subtilis* DnaB is unrelated to *E. coli* DnaB. DnaD is recruited to the origin through an interaction with DnaA, moreover DnaD binding has been shown to be accompanied by pronounced bending of origin DNA. Unwinding of the duplex appears to be assisted by the formation of DnaD scaffolds, which may provide further anchorage points for DnaA (Zhang *et al.*, 2008). DnaB is subsequently recruited, appearing to play a role in helicase loading along with the helicase loader DnaI (Velten *et al.*, 2003).

During rapid growth, bacteria reinitiate replication before the previous cycle of replication is complete, giving rise to multiple replication forks. Daughter cells thus inherit chromosomes that are already undergoing replication. This emphasizes the need for precise mechanisms to control the frequency of initiation of DNA replication so that it matches the frequency of cell division and nutrient availability. Regulatory mechanisms take the form of proteins and *cis*-acting DNA elements which typically act on DnaA or *oriC.* Protein regulators vary between genera. Notably, *B. subtilis* and *E. coli* employ a range of replication regulators that lack known homologues in the other species (*E. coli* Hda, DiaA, SeqA; *B. subtilis* YabA, Soj, SirA, Spo0A) (Katayama *et al.*, 2010; Briggs *et al.*, 2012), reflecting differences in their mechanisms of regulatory control. For example, in *E. coli* Hda inactivates DnaA by promoting ATP hydrolysis in DnaA–ATP, whereas its functional homologue in *B. subtilis*, YabA, acts by both sequestering DnaA at the replication fork, and by inhibiting DnaA oligomerization (Scholefield and Murray, 2013) (Soufo *et al.*, 2008).

An additional specialized DNA replication checkpoint exists in Gram-positive bacteria of the genera *Bacilli* and *Clostridia* during sporulation under conditions of nutrient depletion. Sporulation begins with an asymmetric cell division producing genetically identical daughter cells of unequal size. The larger mother cell and the smaller forespore must each inherit a complete copy of the genome in order to drive the developmental program. Therefore, DNA replication is regulated and monitored at the onset of sporulation to ensure that two intact copies of the chromosome are present in the pre-divisional cell. DnaA contributes to this DNA replication check-point through its role as a transcription factor. In *B. subtilis*, DnaA activates the expression of *sda*, which encodes an inhibitor of the sporulation sensor kinases, KinA and KinB. Sda thus serves to delay sporulation by limiting the phosphorylation of the master sporulation response regulator, Spo0A. Sda is an intrinsically unstable protein whose levels fluctuate with the cell cycle, reaching a minimum immediately prior to the initiation of a new round of DNA replication (Burkholder *et al.*, 2001; Veening *et al.*, 2009). This creates a small ‘window of opportunity’, for a threshold concentration of Spo0A∼P to be achieved and for sporulation to commence.

SirA, a protein produced under Spo0A∼P regulation, has been identified as an inhibitor of DNA replication that plays a specific role in preventing replication re-initiation in cells committed to sporulation (Rahn-Lee *et al.*, 2009). Although single deletion mutants of s*irA*, like those of *sda*, display only mild phenotypes, under conditions of nutrient depletion leading to sporulation *sda*/*sirA* double mutants are severely impaired in chromosome copy number control, indicating a shared role in controlling DNA replication (Veening *et al.*, 2009). Artificial induction of expression of *sirA* in vegetatively growing *B. subtilis* blocks replication and causes cell death in a DnaA-dependent manner (Wagner *et al.*, 2009). Cells artificially induced to sporulate under conditions of rapid growth undergo a marked decrease in chromosome copy number which is partially relieved by deletion of *sirA* (Rahn-Lee *et al.*, 2009). These experiments imply that SirA is an inhibitor of DNA replication during sporulation that acts by binding to DnaA. Furthermore, the SirA binding determinants of DnaA have been mapped to its N-terminal domain, DnaA^DI^ (Rahn-Lee *et al.*, 2011).

SirA has no significant sequence similarity to other proteins besides orthologues in *Bacilli*. Here, we have solved the structure of SirA from *B. subtilis* in complex with DnaA^DI^ providing the first structure of a DnaA domain in an inhibitory complex. The structure reveals a heterodimer with an α-helical interface. The importance of this interface for SirA–DnaA interaction *in vitro* and *in vivo* has been demonstrated by analysis of a panel of site-directed mutants. Furthermore, localization of GFP–SirA within sporulating cells indicates that the protein accumulates at the replisome, likely through a direct interaction with DnaA. Interestingly, the structure reveals a conserved binding site on DnaA^DI^ that is used by DnaA in *H. pylori* and *E. coli* to bind the replication activators HobA and DiaA, respectively, implying this surface is functionally important in DNA replication initiation.

## Results

### Coexpression with DnaA^DI^ confers solubility on recombinant SirA

Attempts to produce recombinant SirA in *E. coli* yielded disappointing results; although SirA could be produced at high levels in a number of *E. coli* expression strains, the protein always partitioned into the insoluble fraction upon cell lysis. Variations in growth conditions or lysis procedures failed to overcome the insolubility of SirA. Following a report that the determinants of SirA binding to DnaA reside in its N-terminal domain (DnaA^DI^) (Rahn-Lee *et al.*, 2011), we generated a coexpression construct in which sequences encoding DnaA^DI^ (fused to an N-terminal cleavable polyhistidine-tag) and SirA were expressed from separate promoters on the same vector. Strikingly, this coexpression strategy led to the appearance of SirA in the soluble fraction following cell lysis, presumably the result of its interaction with DnaA^DI^. Consistent with the notion that recombinant SirA and DnaA^DI^ were forming a complex, the two proteins co-purified. SirA was retained on an immobilized nickel affinity column with His-tagged DnaA^DI^ and co-eluted with the latter. Moreover, the two proteins eluted together following gel filtration chromatography.

### SirA and DnaA^DI^ form a heterodimer

**Figure 1:**
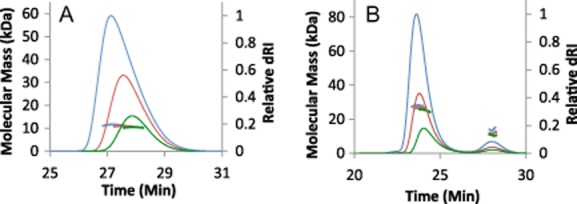
Molecular mass measured from SEC-MALLS analysis. In A and B, the thinner lines trace the differential refractive index of the eluate from a Superdex 10/30 S75 column as a function of time. The thicker lines represent the weight average molecular weight of the species in the eluate, calculated from refractive index and light-scattering measurements. A. Overlay of chromatograms for DnaA^DI^ at 3 concentrations: 1 mg ml^−1^ (green), 2.5 mg ml^−1^ (red) and 5 mg ml^−1^ (blue), revealing species of mass 11 kDa indicating that DnaA^DI^ is a monomer. B. Overlay of chromatograms for SirA–DnaA^DI^ at 3 concentrations: 0.5 mg ml^−1^ (green), 1.0 mg ml^−1^ (red) and 2.5 mg ml^−1^ (blue). The derived M_w_ values for the principal species are 25–28 kDa indicating that the SirA–DnaA^DI^ complex is a 1:1 heterodimer. There is evidently, excess/dissociated DnaA^DI^ giving rise to the minor peak eluting at ∼28 min.

The stoichiometry of the SirA–DnaA^DI^ complex was determined using size-exclusion chromatography with multi-angle laser light scattering (SEC-MALLS). In these experiments, samples are fractionated on a gel-filtration column and the absorbance at 280 nm and the refractive index of the eluate are monitored together with the multi-angle laser light scattering of the sample. This enables the weight average molecular weight (M_w_) of species in the eluate to be calculated continuously. Samples of DnaA^DI^ and SirA–DnaA^DI^ were analysed at a series of protein concentrations. As shown in Fig. 1A, DnaA^DI^ elutes from the size exclusion column as a single A_280_ peak at ∼ 27.5 min and has an experimentally determined molecular mass of ∼ 11 kDa. This suggests that DnaA^DI^ from *B. subtilis* is a monomer (calculated molecular mass = 9.7 kDa) in contrast to *E. coli* DnaA^DI^ which is reported to form dimers under similar conditions (Abe *et al.*, 2007).

The SirA–DnaA^DI^ elution profile has two peaks: a major peak at ∼ 24 min and a minor peak at ∼ 28 min (Fig. 1B). The minor peak comprises 8–12% of the total protein content and the analysis above suggests this is monomeric DnaA^DI^. The major peak (88–92% of the total protein content), which corresponds to a molecular mass of 25–28 kDa, is consistent with a 1:1 heterodimer of SirA : DnaA^DI^ (calculated molecular mass = 28.7 kDa). A discernible shift in the relative sizes of the major and minor peaks occurs as the protein concentration changes in these experiments. There is an increasing area under the minor peak as the protein concentration is lowered, accompanied by a small shift in the elution time associated with the major peak and by a decrease in its associated molecular mass at lower concentrations. This is consistent with increasing complex dissociation at lower protein concentrations. It is clear, however, that under these experimental conditions the SirA–DnaA^DI^ heterodimer is the predominant species.

### The crystal structure of the SirA–DnaA^DI^ complex

The crystal structure of SirA–DnaA^DI^ was solved to 1.7 Å resolution using single anomalous dispersion (SAD) phasing techniques (Table 1). Native and selenomethionine-substituted SirA–DnaA^DI^ crystals grew under different conditions from PEG 3350 containing solutions (see *Experimental procedures*). Although both crystals belong to space group P2_1_, the crystals are different (Table 1). SeMet-derivative crystals contain one complex per asymmetric unit (one molecule of SirA, one molecule of DnaA^DI^) while the asymmetric unit of the native crystals contains two complexes. The SeMet structure was solved and partially refined to allow solution of the native structure by molecular replacement. The refined model encompasses residues 2–141 of SirA (Met^1^ and residues 142–148 being disordered) in both molecules A and C. Residues 1–81 of DnaA^DI^ are defined in molecules B and D (the C-terminal Gln^82^ being disordered). A vestigial N-terminal Gly-Pro-Ala sequence inherited from the DnaA^DI^ polyhistidine-tag can be seen in molecule D, and an additional N-terminal Ala is visible in molecule B. SirA–DnaA^DI^ is seen as a heterodimer, with molecules A (SirA) and B (DnaA^DI^) forming one heterodimer and molecules C and D forming the other. The electron density maps reveal a 2-mercaptoethanol (BME) molecule linked through a disulphide bond to Cys^125^ of both SirA chains in the asymmetric unit (Fig. S1). The presence of this adduct explains species observed in the electrospray ionization mass spectrum of SirA of 18 776 Da, 79 Da larger than that of SirA; 18 697 Da. BME was present during the purification steps as it was found to improve the solubility of the DnaA^DI^–SirA complex.

**Table 1:** X-ray data collection and refinement statistics

	SirA–DnaADI SeMet	SirA–DnaADI Native I
**Data collection**		
X-ray source	DLS, i24	DLS, i03
Wavelength (Å)	0.9789	0.9763
Resolution range (Å)	40.8–2.09	62.76–1.65
Space group	P2_1_	P2_1_
Unit cell parameters		
a, b, c (Å)	51.35, 35.63, 63.27	77.29, 34.69, 84.74
α = γ, β (°)	90, 92.77	90, 102.09
No. of unique reflections^a^	13 549/989	52 893/2598
Completeness (%)^a^	98.7/99.1	98.9/99.6
Redundancy^a^	3.2/3.3	2.8/2.8
*I*/σ(*I*)^a^	11.9/1.9	12.5/1.9
*R*_merge_^b^ (%)^a^	7.4/79.9	3.9/45.8
**Refinement and model statistics**		
Resolution range (Å)		62.84–1.65
*R*-factor^c^ (*R*_free_^d^)		13.1 (19.7)
Reflections (working/*R*_free_)		50172/2706
Outer-shell/high resolution range		1.69–1.65
Outer-shell^e^/high resolution *R*-factor^c^ (*R*_free_)^d^		19.0 (27.9)
Outer-shell/high resolution reflections (working/free)		3677/214
Molecules per asymmetric unit		4
rmsd from ideal geometry^f^		
Bond lengths (Å)		0.017
Bond angles (°)		1.8
Average B-factor (Å2)		27.8
Ramachandran plot^g^		98.16/0.92/0.92

a The first number refers to the overall data set, the second refers the outer resolution shells; Native: 1.68-1.65 Å; SeMet: 2.15-2.09 Å.

b *R*_merge_ = ∑*_hkl_*∑*_i_*|*I_i_* − *< I >* |/∑*_hkl_*∑*_i_* < *I* > where *I_i_* is the intensity of the *i*th measurement of a reflection with indexes *hkl* and *< I >* is the statistically weighted average reflection intensity.

c *R*-factor = ∑||*F_o_*| − |*F*_c_||/∑|*F*_o_| where *F*_o_ and *F*_c_ are the observed and calculated structure factor amplitudes respectively.

d *R*-free is the *R*-factor calculated with 5% of the reflections chosen at random and omitted from refinement.

e Outer shell for refinement corresponds to 1.69–1.65 Å.

f Root-mean-square deviation of bond lengths and bond angles from ideal geometry.

g Percentage of residues in most-favoured/allowed/disallowed regions of the Ramachandran plot.

**Figure 2:**
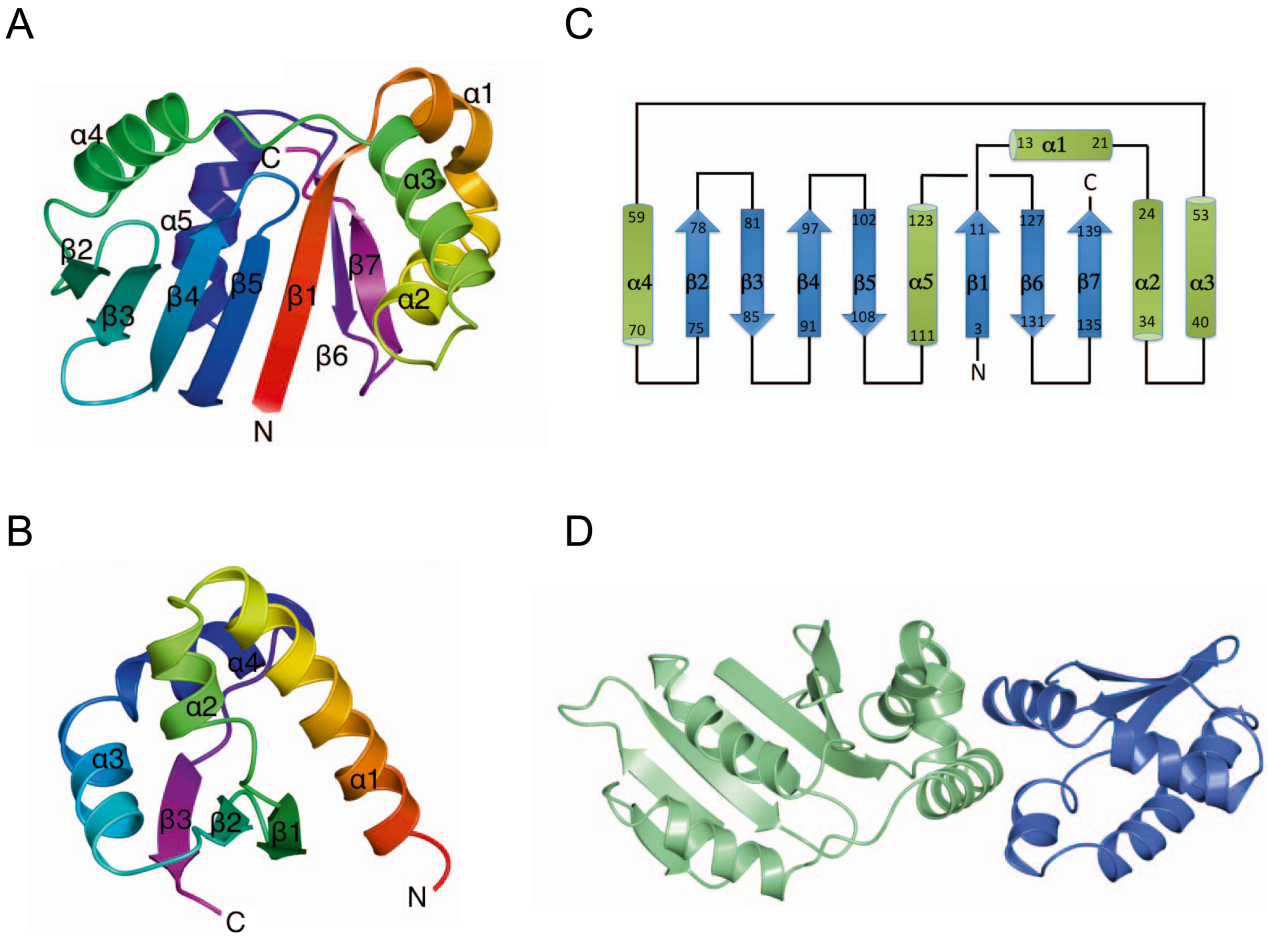
The structure of the SirA–DnaA^DI^ complex. A and B. Ribbon diagram of the SirA (A) and DnaA^DI^ (B) chains from the complex. In each case, the chain is colour-ramped from its N-terminus (red) to the C-terminus (magenta) and the secondary structure elements are labelled. These and subsequent structure figures were produced using the program CCP4mg (McNicholas *et al.*, 2011). C. The polypeptide chain topology in SirA. D. Ribbon diagram of the SirA–DnaA^DI^ complex with SirA shown in light green and DnaA^DI^ shown in blue.

SirA consists of a single globular domain comprising seven β-strands and five α-helices in the order β1-α1-α2-α3-α4-β2-β3-β4-β5-α5-β6-β7 (Fig. 2A and C). The SirA fold consists of a central seven-stranded twisted β-sheet with strand order β2-β3-β4-β5-β1-β6-β7, flanked on either side by two α-helical regions, one comprising helices α1, α2 and α3 and the other of helices α4 and α5. Comparative analysis of the SirA chain topology using PDBeFold identified the kinase associated domain 1 from the protein KCCP4 (PDB entry 3osm) as the highest Q-scoring hit with 79 Cα atoms overlaying with a positional root mean squared deviation of 2.4 Å. This domain has been identified as a membrane association domain that binds acidic phospholipids (Moravcevic *et al.*, 2010). The region of structural similarity spans residues 2–9 and 53–124 covering the β1-α4-β2-β3-β4-β5-α5 segment. The other highest scoring matches, the core domain of the human ribosomal protein L10 (2pa2) and the yeast mitochondrial protein frataxin (4ec2), exhibit structural similarity to the same region of SirA.

DnaA^DI^ from *B. subtilis* (Fig. 2B) consists of four alpha helices and three beta strands in the order α1-α2-β1-β2-α3-α4-β3 with a β-sheet topology of β1-β2-β3. It has a closely similar topology to the previously determined structures of the corresponding domains of DnaA from *E. coli* (Abe *et al.*, 2007), *M. genitalium* (Lowery *et al.*, 2007) and *H. pylori* (Natrajan *et al.*, 2009)*.* Thus, it shares the K homology domain motif that is widespread in single-stranded nucleic acid binding proteins.

### The SirA–DnaA^DI^ interface

**Figure 3:**
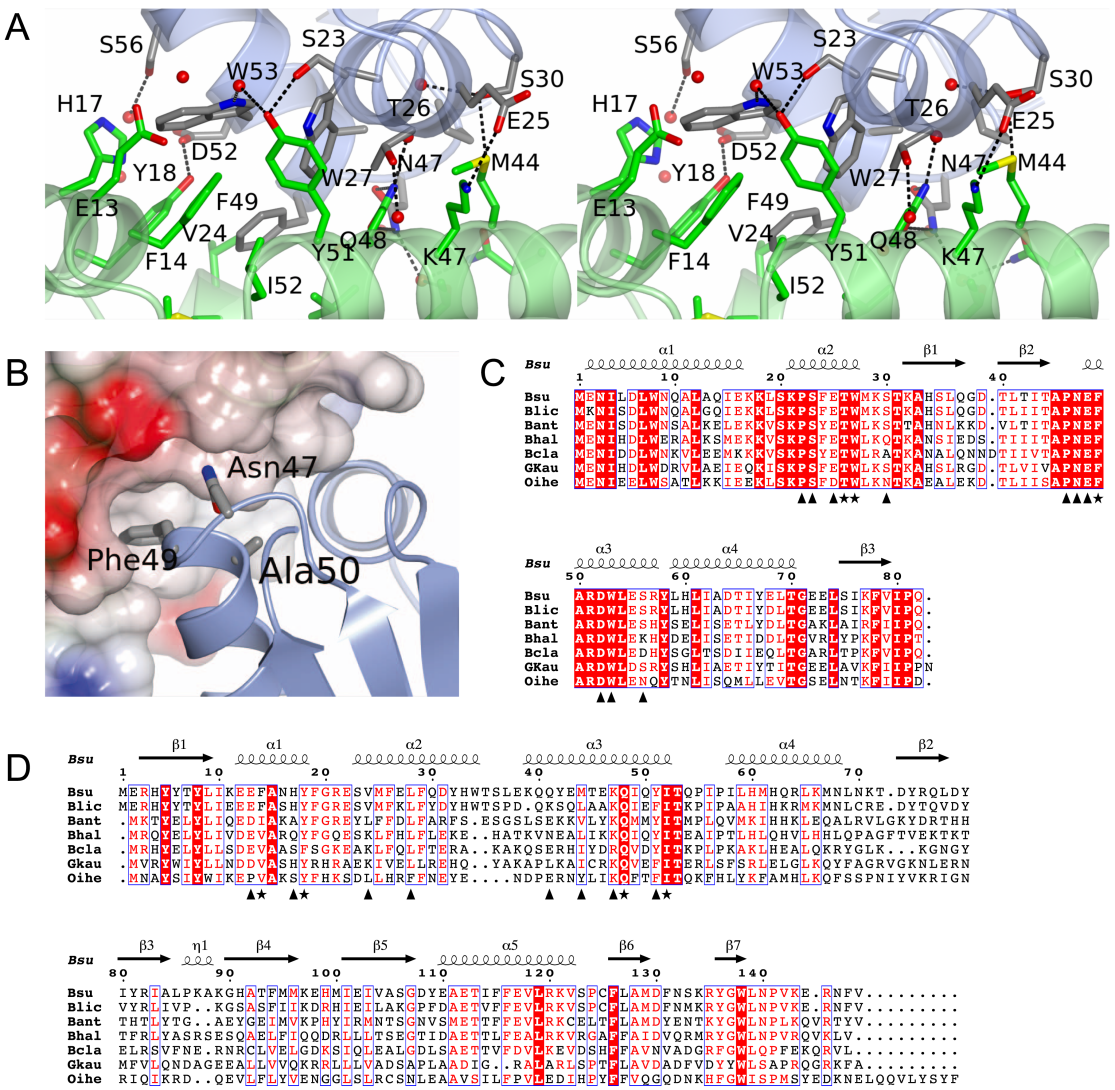
The SirA–DnaA^DI^ interface. A. Stereoview of the complex between DnaA^DI^ (chain D) and SirA (chain C) represented as light blue and light green ribbons, respectively. Side-chains of labelled residues are displayed in cylinder format and coloured by atom type with nitrogen (blue), oxygen (red), sulphur (yellow) and carbons coloured in grey for DnaA^DI^ and green for SirA. Water molecules are represented as red spheres, and polar interactions are denoted by dashed lines. B. Mapping onto the structure of DnaA^DI^ the sites corresponding to mutations in *dnaA* that allow growth of *B. subtilis* even when *sirA* is being overexpressed (Rahn-Lee *et al.*, 2011). SirA is rendered as a partially transparent electrostatic surface and DnaA^DI^ as a ribbon with the side-chains of residues Asn47, Phe49 and Ala50 in cylinder format. C and D. Alignment of the sequences of orthologues of DnaA^DI^ (C) and SirA (D) from selected *Bacillus* species; Bsu, *B. subtilis*; Blic, *B. licheniformis*; Bant, *B. anthracis*; Bhal, *B. halodurans*; Bcla, *B. clausii*; Gkau, *Geobacillus klaustophilus*; Oihe, *Oceanobacillus iheyensis.* Symbols below the alignments indicate interfacial residues in the respective molecules contributing to the core (asterisks) and the rim (triangles). Secondary structure elements and residue numberings are displayed above the alignment. The images were created using ESPript (Gouet, 2003).

The binding of SirA and DnaA^DI^ is mediated by helices α1, α2 and α3 of SirA and α2 and α3 of DnaA^DI^ (Figs 2D and 3A). As an extensively α-helical interface the interactions of the two proteins are dominated by side-chain–side-chain contacts (Fig. 3A). Seventeen residues on each chain contribute to the interface which constitutes 12% and 8% of the surface areas of DnaA^DI^ and SirA, respectively. In the complex, 1240 Å^2^ of otherwise accessible surface area is buried in the interface. This buried surface area is at the lower end of the range observed in non-obligate dimeric protein-protein complexes (Janin *et al.*, 2008). In the SirA binding surface of DnaA^DI^, Thr26, Trp27 and Phe49 contribute to the core of the interface with residues Pro22, Ser23, Glu25, Ser30, Pro46, Asn47, Glu48, Asp52, Ser56 and Trp53 prominent in the rim. As shown in Fig. 3C, these residues are very strongly conserved in a set of DnaA orthologues from endospore-forming bacteria. On the corresponding DnaA^DI^ binding surface of SirA, Phe14, Tyr18, Gln48 and Ile52 contribute to the core and Glu13, His17, Val24, Leu28, Gln41, Met44, Lys47 and Tyr51 are prominent in the rim. Again core residues are well conserved with some variation observed in the residues constituting the rim (Fig. 3D).

The SirA binding determinants of DnaA have previously been explored using genetic approaches. Induction of *sirA* expression under nutrient rich conditions inhibits growth of *B. subtilis*. Four strains were identified which harbour mutations in *dnaA* that were able to suppress this slow growth phenotype accompanying *sirA* induction (Rahn-Lee *et al.*, 2011). Analysis of the sequence of these *dnaA* alleles revealed point mutations giving rise to Asn47Asp, Phe49Tyr, Ala50Val and Ala50Thr substitutions (Rahn-Lee *et al.*, 2011). Yeast two hybrid analysis confirmed that mutations at these residues prevent DnaA from interacting with SirA, suggesting they affect SirA–DnaA^DI^ complex formation (Rahn-Lee *et al.*, 2011). The SirA–DnaA^DI^ structure reveals that Asn47 and Phe49 make direct interactions with SirA (Fig. 3A and B). Asn47 of DnaA forms a pair of hydrogen bonds with Gln48 on helix α3 of SirA, while the side-chain of Phe49 of DnaA projects into a hydrophobic pocket created by helices α1, α2 and α3 of SirA. In contrast, Ala50 does not contact SirA in the complex; instead it is buried within DnaA^DI^ in such a way that it determines the structure of the interface (Fig. 3B). It is expected that mutations at this position which introduce bulkier side-chains, such as valine or threonine, will alter the structure of the interaction surface leading to lower affinity binding of SirA. In summary, the structure of SirA–DnaA^DI^ confirms previous interpretations of the genetic data.

### Substitutions at the SirA–DnaA^DI^ interface affect the SirA–DnaA interaction *in vitro*

To confirm the importance of the protein-protein interface observed in the SirA–DnaA^DI^ crystals, we assayed the interactions of site-directed mutants of *sirA* and *dnaA^DI^ in vitro*. Mixing experiments using the purified proteins are not possible because we are unable to produce soluble SirA in the absence of coexpression with DnaA^DI^. Instead we took advantage of the dependence of SirA solubility on its co-production with, and binding to, DnaA^DI^ in developing a qualitative binding assay. We hypothesized that disruption of the interaction between SirA and DnaA^DI^ following coexpression would reduce or abolish SirA solubility.

Site-directed mutagenesis was used to introduce alanine substitutions into the pET-YSBLIC3C-DnaA^DI^ SirA coexpression vector at codons that specify the SirA–DnaA^DI^ interface in the crystal structure. Three alanine substitutions were introduced into SirA: Phe14Ala, Tyr18Ala and Gln48Ala. These residues contribute 45 Å^2^, 45 Å^2^ and 80 Å^2^ of buried surface area respectively to the interface with the phenolic hydroxyl of Tyr18 forming a charge-dipole interaction with Asp52 of DnaA and the side-chain amide of Gln48 forming a pair of hydrogen bonds with the amide of Asn47 of DnaA (Fig. 3A). A further three substitutions were introduced into DnaA^DI^, these being Thr26Ala, Trp27Ala and Phe49Ala. Thr26, Trp27 and Phe49 contribute 75 Å^2^, 50 Å^2^ and 135 Å^2^ of surface area respectively to the interface. After sequencing to confirm the presence of the mutations, the mutated plasmids were introduced into *E. coli* BL21 and expression experiments were performed. The solubility of the recombinant proteins was compared to that of the wild-type proteins by SDS-PAGE of cell fractions following lysis (Fig. S2).

**Figure 4:**
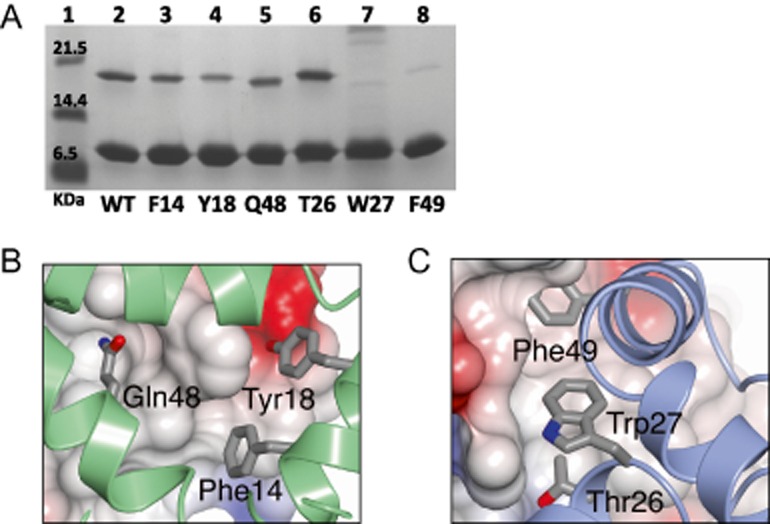
The SirA–DnaA^DI^ interface analysed by site-directed mutagenesis. A. SDS-PAGE. Cultures of cells harbouring plasmids encoding wild type and alanine-substituted variants of His-tagged DnaA^DI^ and SirA were grown. Soluble cell lysates were prepared and loaded onto a Ni-NTA column. High imidazole eluate fractions were collected for analysis. Samples of the eluate fractions containing approximately normalized levels of DnaA^DI^ were loaded so that the efficiency of SirA pull-down could be compared. Lane 1 contains molecular weight markers. Lane 2: wild type DnaA^DI^–SirA. Lanes 3–5: Native DnaA^DI^ and the SirA variants, loaded as follows; Lane 3: SirA(F14A), Lane 4: SirA(Y18A), Lane 5: SirA(Q48A). Lanes 6–8: Samples of native SirA and the DnaA^DI^ variants, loaded as follows; Lane 6: DnaA^DI^(T26A), Lane 7: DnaA^DI^(W27A), Lanes 8: DnaA^DI^(F49A). B and C. Core residues from the DnaA ^DI^-interacting surface of SirA (B) and the SirA interacting surface of DnaA^DI^ (C) which were sites of alanine substitution. (B) DnaA^DI^ is shown as an electrostatic surface with SirA represented as a green ribbon with the side-chains of F14, Y18 and Q48 displayed as cylinders. (C) SirA is shown as an electrostatic surface with DnaA^DI^ represented as a blue ribbon with the side-chains of T26, W27 and F49 displayed as cylinders.

These experiments show that there are reduced levels of SirA in the soluble lysate fractions (labelled S in Fig. S2) of cells producing SirA^F14A^ and SirA^Y18A^ and negligible levels of SirA in these fractions from cells producing DnaA^DI,W27A^ and DnaA^DI,F49A^. This suggests weaker binding of SirA by DnaA^DI^. However, interpretation of these experiments is complicated by variability in the levels of DnaA^DI^ present in these fractions. Thus, the effect of each mutation on the interaction of SirA with DnaA^DI^ was further probed using a pull down assay where the soluble fraction of the cell lysate was loaded onto a Ni-affinity column. The latter was washed extensively with loading buffer and bound proteins were eluted in a buffer containing a high concentration of imidazole. pET-YSBLIC3C-DnaA^DI^SirA directs expression of DnaA^DI^ with a hexahistidine tag together with untagged SirA. Thus, any retention of SirA is expected to result from its interaction with the histidine-tagged DnaA^DI^. The eluate (E) fractions shown in Fig. S2 were diluted to normalize to an approximately equivalent amount of DnaA^DI^ and the samples again resolved by SDS-PAGE with Coomassie staining. As can be seen in Fig. 4A, the DnaA^DI,W27A^ and DnaA^DI,F49A^ mutations have the most striking effect, with little discernable SirA eluted from the Ni-NTA column in the high imidazole fraction. In marked contrast, DnaA^DI,T26A^ supports wild-type levels of SirA recovery after the nickel pull down. For the three SirA mutant proteins, the effects are more modest. Quantification of the DnaA^DI^ and SirA band intensities in Fig. 4A using the software ImageJ revealed, relative to the wild type SirA, 1.5-fold lower recovery of SirA^F14A^ and SirA^Q48A^ and a 2.5-fold lower recovery of SirA^Y18A^.

Collectively, these results correlate with the SirA–DnaA interface in the crystal structure. Residues Phe14, Trp18 and Gln48 of SirA form contacts with DnaA^DI^ which would be weakened upon truncation of these side-chains to alanine (Fig. 4B). The side-chains of residues Trp27 and Phe49 in DnaA^DI^ project away from the surface of the protein into a hydrophobic groove on the SirA surface, forming extensive van der Waals contacts across the SirA–DnaA interface (Fig. 4C). The results indicate that truncation of either of these large hydrophobic residues strongly affects the SirA–DnaA interaction due to the loss of these contacts. By contrast, Thr26 of DnaA^DI^ binds at the edge of a hydrophobic groove and although it is largely buried, its hydroxyl is able to form a hydrogen bond to a recessed water molecule on the protein surface. Moreover, substitution of threonine with alanine is a less drastic change, evidently allowing DnaA^DI, T26A^ to maintain its interaction with SirA.

### GFP–SirA colocalizes with the replisome in sporulating cells

**Figure 5:**
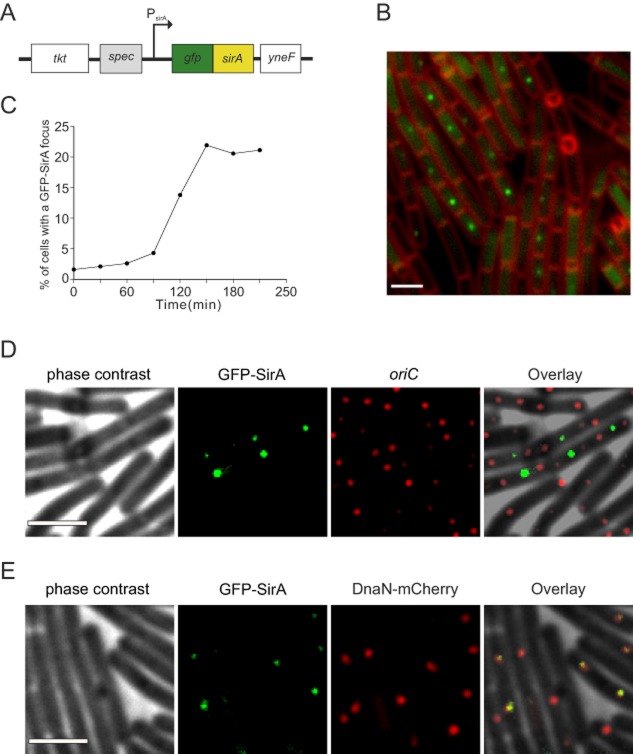
Localization of GFP–SirA *in vivo*. A. Schematic diagram showing the modified *sirA* locus used for localization studies. Chromosomal *sirA* was replaced with *gfp–sirA* under the control of its native expression system. B. GFP–SirA localization during sporulation of *B. subtilis* 150 min post resuspension in starvation media. Membrane dye FM5-95 was used to highlight the outlines of the cells. Scale bar = 3 μm. *gfp–sirA* (NR3). C. Temporal analysis of GFP–SirA foci formation during sporulation. At least 500 cells were analysed at each time-point. D. Colocalization of GFP–SirA with *oriC* during sporulation (150 min post resuspension in starvation media). More than 200 cells were analysed and a representative image is shown. Scale bar = 3 μm. *gfp–sirA oriC^tetO^*^/TetR−mCherry^ (NR164). E. Colocalization of GFP–SirA with the replisome during sporulation (150 min post resuspension in starvation media). More than 100 cells were analysed and a representative image is shown. Scale bar = 3 μm. *gfp–sirA dnaN–mCherry* (NR168).

The biochemical analysis of SirA–DnaA^DI^ complex formation provides strong evidence that the interaction observed in the crystal structure is also formed between the two proteins in solution. To study the physiological relevance of the proposed SirA–DnaA interface, SirA activity was examined *in vivo*. Visualization of SirA was achieved by replacing the endogenous gene with *gfp–sirA* (expressed from its native transcriptional and translational regulatory sequences; Fig. 5A), inducing cells to sporulate by nutrient deprivation, and detecting the localization of GFP–SirA using epifluorescence microscopy (Fig. 5B). A time-course experiment showed that GFP–SirA foci began to appear approximately 90 min after cells were resuspended in starvation medium. By 150 min, the number of cells containing a GFP–SirA focus reached its maximum (∼ 20%; Fig. 5C). In the majority of cases (> 80%) GFP–SirA foci were located near mid-cell. Previous studies suggested that SirA inhibits new rounds of DNA replication by inhibiting the binding of DnaA to the origin of replication (Wagner *et al.*, 2009). However, the mid-cell localization of GFP–SirA foci is contrary to origin positioning in sporulating cells where the two origins are positioned towards the cell poles, suggesting that SirA was not accumulating at *oriC*.

In order to further investigate GFP–SirA localization we constructed a strain that allowed visualization of both origin regions and SirA. An array of *tet* operators was inserted near *oriC* and the Tet repressor was fused to a red fluorescent protein (TetR-mCherry); interaction of TetR-mCherry with the *tetO* array produces a fluorescent focus near each origin of replication. Cells were induced to sporulate by nutrient deprivation and the localization of GFP–SirA was determined in respect to the origin regions. In the majority of cells with a GFP–SirA focus there was no colocalization of SirA with *oriC* (78% with non-overlapping signals; Fig. 5D)*.* Only 8% of the GFP–SirA foci appeared to colocalize with the origin regions, with the remaining 14% of cells containing a GFP–SirA focus that partially overlapped with the origin marker (Fig. 5D). These results show that during sporulation GFP–SirA mainly accumulates away from the replication origin.

A previous study in *B. subtilis* reported that DnaA colocalizes with the replisome at mid-cell via the YabA–DnaN complex during DNA replication (Soufo *et al.*, 2008). We hypothesized that SirA might be interacting with DnaA when it is bound to the replication machinery. To begin testing this model we examined GFP–SirA localization in cells that contained a fusion of a red fluorescent protein to the sliding clamp of the replisome (DnaN–mCherry). Cells were induced to sporulate by nutrient deprivation and the localization of GFP–SirA was determined in respect to the replisome. Strikingly, in cells containing a GFP–SirA focus the vast majority colocalized with DnaN–mCherry (88%; Fig. 5E). This result indicates that GFP–SirA accumulates at the replisome.

### Substitutions at the SirA–DnaA^DI^ interface affect GFP–SirA localization *in vivo*

**Figure 6:**
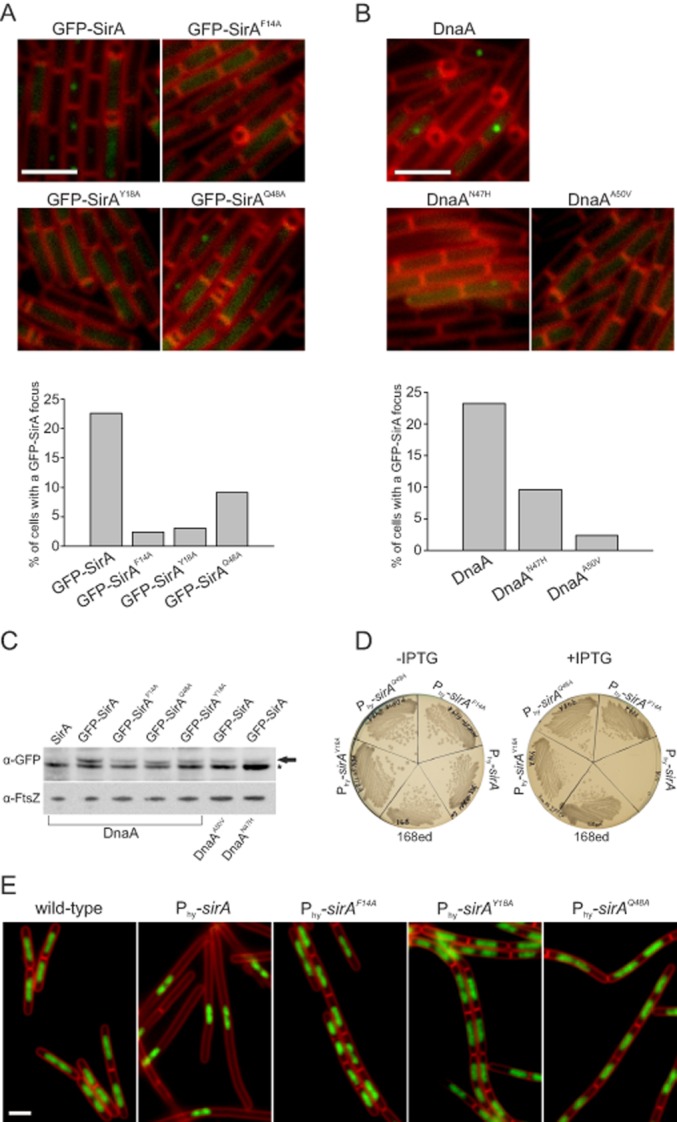
Examination of the SirA–DnaA interface *in vivo*. A. Amino acid substitutions in SirA inhibit GFP–SirA foci formation. For each strain over 700 cells were analysed and the experiment was repeated at least three times. Quantification of a representative dataset is shown below. Scale bar = 3 μm. *gfp–sirA* (NR3); *gfp–sirA^F14A^* (NR130); *gfp–sirA^Y18A^* (NR156); *gfp–sirA^Q48A^* (NR131). B. Amino acid substitutions in DnaA inhibit GFP–SirA foci formation. For each strain over 700 cells were analysed and the experiment was repeated at least three times. Quantification of a representative dataset is shown below. Scale bar = 3 μm. *gfp–sirA* (NR3); *gfp–sirA dnaA^A50V^* (NR5); *gfp–sirA dnaA^N47H^* (NR154). C. Immunoblot analysis showing levels of GFP-tagged SirA proteins. Cell samples were taken 150 min post resuspension in starvation media. The arrow points to GFP–SirA, the star highlights a contaminating band. Immunoblot of FtsZ was utilized to standardize the amount of protein from different samples. Wild-type (168ed); *gfp–sirA* (NR3); *gfp–sirA^F14A^* (NR130); *gfp–sirA^Y18A^* (NR156); *gfp–sirA^Q48A^* (NR131); *gfp–sirA dnaA^A50V^* (NR5); *gfp–sirA dnaA^N47H^* (NR154). D. Wild-type and mutant *sirA* were placed under control of an IPTG-inducible promoter and streaked on nutrient agar plates in the presence and absence of IPTG (3 mM). Wild-type (168ed); P*_hyperspank_*-*sirA* (NR171); P*_hyperspank_*-*sirA^F14A^* (NR172); P*_hyperspank_*-*sirA^Q48A^* (NR173); P*_hyperspank_*-*sirA^Y18A^* (NR174). E. The effects of overexpressing various SirA proteins at the single cell level were studied by growing cells in liquid CH medium. The images were taken 180 min after induction of gene expression with IPTG (3 mM). Membrane dye FM5-95 was used to highlight the outlines of the cells, DAPI was used to stain the DNA. Scale bar = 3 μm. Wild-type (168ed); P*_hyperspank_*-*sirA* (NR171); P*_hyperspank_*-*sirA^F14A^* (NR172); P*_hyperspank_*-*sirA^Q48A^* (NR173); P*_hyperspank_*-*sirA^Y18A^* (NR174).

To investigate whether the localization of GFP–SirA at the replisome was dependent upon an interaction with DnaA, first the wild-type *sirA* (from the *gfp–sirA* chimera) was replaced with *sirA* mutants altering the residues identified in the SirA–DnaA^DI^ structure implicated in complex formation (*gfp–sirA^F14A^*, *gfp–sirA^Y18A^* or *gfp–sirA^Q48A^*) and the localization GFP–SirA proteins was determined during sporulation. All of the *sirA* mutants caused a significant decrease in the number of cells containing a fluorescent focus (Fig. 6A). Both *gfp–sirA^F14A^* and *gfp–sirA^Y18A^* mutants reduced the number of GFP foci to background levels (i.e. – in the absence of a GFP fusion), while the *gfp–sirA^Q48A^* mutant decreased foci formation 2.5-fold. These results indicate that the amino acid residues in SirA identified in the structure at the interface with DnaA^DI^ are required for GFP–SirA localization at the replisome.

Next we attempted to replace *dnaA* with *dnaA^T26A^*, *dnaA^W27A^* and *dnaA^F49A^*; however, we were unable to isolate any of these mutants (see *Discussion*). In contrast, mutations in *dnaA* at locations that were previously shown to inhibit SirA activity *in vivo* (*dnaA^A50V^* and *dnaA^N47H^*) could be readily generated; therefore, GFP–SirA localization was determined using these *dnaA* alleles. Figure 6B shows that both DnaA variants inhibited GFP–SirA foci formation, with DnaA^A50V^ reducing foci formation to background levels and DnaA^N47H^ decreasing foci formation 2.4-fold. Immunoblot analysis of GFP-SirA proteins showed similar levels in all mutants tested, indicating that the absence of foci formation was not due to altered protein expression (Fig. 6C). Taken together with the analysis of the *sirA* mutants, these results show that the SirA–DnaA^DI^ interface identified in the crystal structure is critical for GFP–SirA localization *in vivo* and they suggest that GFP–SirA localization is mediated through a direct interaction with replisome-bound DnaA.

### Substitutions at SirA–DnaA^DI^ interface render cells resistant to lethal effects of SirA overexpression in vegetatively growing cells

We attempted to determine whether GFP–DnaA colocalized with the replisome during sporulation, but unfortunately the previously published *gfp–dnaA* reporter strain displayed a severe sporulation defect (Soufo *et al.*, 2008). Therefore, to test whether SirA variants that displayed decreased foci formation were also defective in DnaA regulation, SirA proteins were overexpressed. Wild-type and mutant *sirA* genes were placed under the control of an IPTG-inducible promoter (P*_hyperspank_*) integrated at an ectopic locus. Induction of wild-type SirA inhibited cell growth on solid media, in contrast to the SirA variants (SirA^F14A^, SirA^Y18A^, SirA^Q48A^; Fig. 6D). Induction of wild-type SirA during vegetative growth in liquid media inhibited DNA replication, producing elongated cells that contained a single nucleoid (Fig. 6E). Induction of SirA variants did not affect DNA distribution or cell morphology, and these cells were indistinguishable from a control strain lacking the ectopic *sirA* construct (Fig. 6E). These results show that amino acid residue substitutions in SirA that impair protein localization also affect the ability of SirA to inhibit DnaA activity.

### A conserved binding site on DnaA^DI^

**Figure 7:**
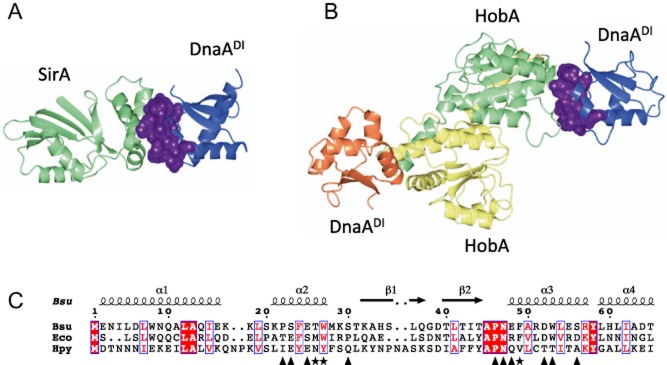
Comparison of *B. subtilis* SirA–DnaA^DI^ with *H. pylori* HobA-DnaA^DI^. A. Ribbon diagram of the *B. subtilis* SirA–DnaA^DI^ complex with SirA shown in light green and DnaA^DI^ shown in blue. B. Ribbon diagram of the *H. pylori* HobA-DnaA^DI^ complex (PDB id code: 2wp0) with two molecules of HobA shown in yellow and light green, and two molecules of DnaA^DI^ shown in blue and coral. The SirA and HobA binding surfaces on DnaA^DI^ in (A) and (B) respectively are shown in purple. C. Alignment of DnaA^DI^ from *B. subtilis*, *E. coli* and *H. pylori.* Symbols below the alignments indicate interfacial residues on DnaA^DI^ in the SirA–DnaA^DI^ structure contributing to the core (asterisks) and the rim (triangles). Secondary structure elements and residue numberings are displayed above the alignment. The images were created using ESPript (Gouet, 2003).

The structure of the SirA–DnaA^DI^ complex and that formed between DnaA^DI^ from *H. pylori* and HobA (Natrajan *et al.*, 2009), a regulator of DNA replication in this pathogen, were compared with each other (Fig. 7). It is apparent that HobA and SirA bind to the same structural site on DnaA^DI^, burying equivalent surface residues (Fig. 7A and B). This is surprising given the divergent effects on DnaA exerted by SirA and HobA. HobA is an essential stimulator of replication initiation in *H. pylori*, in contrast to SirA which is a replication inhibitor. Thus, HobA and SirA achieve opposing regulatory functions by binding to the same structural site on DnaA^DI^. Despite their close tertiary structural correspondence, residues on the regulatory protein binding site on DnaA^DI^ are poorly conserved between *H. pylori* and *B. subtilis*, perhaps reflecting a divergence in their respective regulatory mechanisms (Fig. 7C). Nevertheless, this indicates an important structural site on DnaA^DI^ for the regulation of replication initiation.

## Discussion

The initiator of bacterial DNA replication, DnaA, is stringently regulated so that DNA replication is co-ordinated with cell growth and differentiation. Five negative regulators of DNA replication have been identified in *B. subtilis:* SirA (Rahn-Lee *et al.*, 2009), YabA (Noirot-Gros *et al.*, 2006), Spo0A (Castilla-Llorente *et al.*, 2006) Soj (Murray and Errington, 2008) and DnaD (Bonilla and Grossman, 2012). YabA, Soj and DnaD bind to domain III of DnaA and are thought to block the assembly of helical DnaA filaments at *oriC* (Cho *et al.*, 2008; Scholefield *et al.*, 2012; Scholefield and Murray, 2013). Phosphorylated Spo0A binds to a set of Spo0A-boxes at *oriC* which overlap with DnaA-boxes, suggesting that Spo0A∼P occludes DnaA from the replication origin (Boonstra *et al.*, 2013). SirA is distinct and represents the first, and so far only, *B. subtilis* regulator that interacts with domain I of DnaA (Rahn-Lee *et al.*, 2011). In other organisms however, regulators have been identified which interact with DnaA^DI^ namely, *E. coli* DiaA (Keyamura *et al.*, 2009) and Hda (Su'etsugu *et al.*, 2013), and *H. pylori* HobA (Natrajan *et al.*, 2009). For *E. coli*, DnaA^DI^ has also been shown to interact with the DNA helicase, DnaB (Sutton *et al.*, 1998; Seitz *et al.*, 2000), and to play a role in the oligomeriation of DnaA (Weigel *et al.*, 1999; Felczak *et al.*, 2005), forming dimers *in vitro* (Abe *et al.*, 2007)*.* Here we have elucidated the structure of the SirA–DnaA^DI^ from *B. subtilis* revealing DnaA^DI^ bound in an inhibitory complex. This structure complements that of HobA-DnaA^DI^ from *H. pylori* in which DnaA^DI^ is bound in a complex that leads to activation (Natrajan *et al.*, 2009).

As previously inferred (Rahn-Lee *et al.*, 2011), SirA binds to a site on DnaA^DI^ that corresponds closely to that bound by the regulators HobA from *H. pylori* (Natrajan *et al.*, 2009) and DiaA from *E. coli* (Keyamura *et al.*, 2009)*.* HobA and DiaA are structural homologues which form tetramers that promote DnaA oligomerization and activate the initiation of DNA replication (Zawilak-Pawlik *et al.*, 2011). Each HobA/DiaA tetramer binds to four DnaA^DI^ molecules in a way that is thought to facilitate DnaA-binding to the array of DnaA-boxes distributed at *oriC* (Natrajan *et al.*, 2009). In marked contrast, SirA binds a single molecule of DnaA^DI^ and inhibits DNA replication initiation. Although SirA and HobA/DiaA have quite different three dimensional structures, each buries a structurally equivalent site on DnaA^DI^. It is intriguing therefore that this elicits different regulatory outcomes.

It has been previously proposed that SirA inhibits DnaA binding to *oriC*, based on the observations that SirA disrupts DnaA–GFP localization at *oriC* during vegetative growth, and that there is a SirA-dependent decrease in the amount of DnaA at *oriC* following artificial induction of sporulation (Wagner *et al.*, 2009; Rahn-Lee *et al.*, 2011). At first glance, SirA may achieve this by inhibiting DnaA-oligomerization at *oriC*, since domain I fragments of *E. coli* DnaA form dimers *in vitro*, and the dimerization surface has been identified (Felczak *et al.*, 2005). However, the corresponding surface in *B. subtilis* DnaA^DI^ is located on the opposite side of DnaA^DI^ to the SirA binding surface so that SirA binding would not be expected to prevent dimer formation. Furthermore, we did not observe dimers or oligomers of *B. subtilis* DnaA^DI^ *in vitro.* Thus, it seems unlikely that SirA influences DnaA assembly simply by inhibiting DnaA^DI^-DnaA^DI^ interactions.

Our localization studies indicate that SirA accumulates away from *oriC* and with the replisome near mid-cell during sporulation. We hypothesize that SirA could interact with replisome-bound DnaA to stabilize replisome–DnaA complexes, thereby inhibiting DnaA rebinding at the origin. This is reminiscent of a DnaA-tethering model proposed for YabA (Soufo *et al.*, 2008). Alternatively, our finding that mutations directing alanine substitutions of three residues on the SirA binding surface of DnaA could not be introduced into *dnaA* suggests that SirA could act by inhibiting a critical interaction of DnaA with other components of the initiation complex. In *E. coli*, DnaA^DI^ is implicated directly in the recruitment of the helicase to the nascent replicative complex (Abe *et al.*, 2007). There is no evidence for a DnaA-helicase interaction in *B. subtilis* however, in which helicase recruitment involves additional DNA remodelling proteins (Zhang *et al.*, 2005). DnaD forms multimeric scaffolds on the DNA and recruits DnaB, which in turn is thought to bridge an interaction with the helicase–helicase loader (Zhang *et al.*, 2008; Smits *et al.*, 2010). Thus, we speculate that SirA may inhibit a DnaA–DnaD interaction arresting assembly of the initiation complex.

In summary, this work has defined the interaction surfaces of SirA and DnaA^DI^ and the stoichiometry of their complex. Moreover, we have shown that their interaction is required for GFP-SirA foci formation at the replisome in sporulating *B. subtilis*. These observations will help elucidate the mechanism of action of SirA, the understanding of which is currently limited by imprecise knowledge of the function of domain I of DnaA in this organism.

## Experimental procedures

### Cloning

DNA fragments encoding SirA and domain I of DnaA (DnaA^DI^) were amplified from *B. subtilis* genomic DNA by the polymerase chain reaction (PCR) and inserted into the expression vector pET-YSBLIC3C (Fogg and Wilkinson, 2008) using a ligation independent cloning method. Two constructs were created: one encoding SirA with an N-terminal, 3C protease cleavable His-tag (pET-YSBL3C-SirA) and the other a duet construct containing fragments encoding DnaA^DI^ and SirA cloned upstream and downstream respectively of a LIC Duet Minimal Adaptor (Novagen). The recombinant plasmid (pET-YSBLIC3C-DnaA^DI^SirA) directs the expression of DnaA^DI^ fused to a 3C protease cleavable N-terminal His-tag (His–DnaA^DI^) and SirA from separate bacteriophage T7 promoters. Alanine substitution mutations were introduced into pET-YSBLIC3C-DnaA^DI^SirA by site-directed mutagenesis. The sequences of oligonucleotides used for the cloning and subsequent site-directed mutagenesis of pET-YSBLIC3C-DnaA^DI^SirA are listed in Tables S1 and S2 respectively.

### Expression

The plasmid pET-YSBLIC3C-DnaA^DI^SirA was introduced into *E. coli* BL21 (DE3) cells for the co-overproduction of SirA and His-tagged DnaA^DI^. Overnight cell cultures were used to inoculate 500 ml of Luria–Bertani (LB) media supplemented with 30 μg ml^−1^ kanamycin. Cultures were grown to an OD_600_ of 0.6–0.9 at 37°C with shaking at 180 rpm before protein production was induced with 1 mM isopropyl-β-d-1-thiogalactoside (IPTG). Following induction, cultures were grown at 37°C (180 rpm shaking) for a further 4 h before cells were harvested by centrifugation. SirA/DnaA^DI^ proteins harbouring site-directed mutations were produced in an analogous manner.

For SeMet substituted protein production, overnight cultures of *E. coli* BL21 (DE3) harbouring pET-YSBLIC3C-DnaA^DI^SirA were used to inoculate 500 ml minimal media supplemented with 30 μg ml^−1^ kanamycin. Cultures were grown to an OD_600_ of 0.6–0.8 at 37°C (180 rpm shaking) prior to the addition of an amino acid mixture (50 mg lysine, 50 mg phenylalanine, 50 mg threonine, 25 mg isoleucine, 25 mg leucine, 25 mg valine) to suppress methionine production (Doublié, 1997), and 30 mg selenomethionine. Cultures were grown at 37°C (180 rpm shaking) for a further 15 min prior to induction of recombinant protein production with 1 mM IPTG. Cultures were subsequently grown at 30°C (180 rpm shaking) overnight (16–20 h) before cells were harvested by centrifugation.

### DnaA^DI^–SirA purification

The protein purification procedure was identical for native and SeMet substituted proteins. Harvested cells were resuspended in 50 mM Tris pH 8.5, 200 mM KCl, 20 mM imidazole and 10 mM BME, and an EDTA-free protease inhibitor cocktail tablet (Roche) was added. Resuspended cells were lysed by sonication and the lysate clarified by centrifugation. The cell lysate was loaded on to a His Trap FF crude Ni-affinity column (GE Healthcare) and bound protein eluted with an increasing imidazole concentration gradient (20–500 mM). This step was followed by size-exclusion chromatography on a HiLoad 16/60 Superdex S75 column (GE Healthcare) equilibrated with 50 mM Tris pH 8.5, 200 mM KCl, 10 mM BME. The chromatogram exhibited two protein peaks. SDS-PAGE analysis of the peak fractions showed the earlier eluting peak corresponded to the SirA : His–DnaA^DI^ complex with the later eluting peak containing His–DnaA^DI^ which is produced in excess in the duet expression system. The protein complex and DnaA domain I fractions were combined separately and the N-terminal histidine tag was removed from DnaA^DI^ in both cases by incubation with 3C protease overnight (protease : protein ratio of 1:50). Passage through a second Ni-affinity column to remove the histidine tag and protease yielded pure protein in a buffer of 50 mM Tris pH 8.5, 200 mM KCl, 10 mM BME. The proteins were judged to be pure according to Coomassie staining of SDS-polyacrylamide gels.

### Crystallization and structure solution

Native crystals of SirA–DnaA^DI^ were grown in hanging-drops containing a 1:1 ratio of concentrated protein solution and reservoir solution. Native crystals suitable for data collection were obtained following mixing of a protein solution of 6.4 mg ml^−1^ and a reservoir solution of 100 mM HEPES pH 7.5, 200 mM NH_4_ acetate, 25% (w/v) PEG 3350, 1% (v/v) DMF. Crystals were transferred to a cryoprotectant solution consisting of the reservoir solution containing 20% (v/v) glycerol before being cryocooled in liquid nitrogen. X-ray diffraction data were collected to 1.7 Å resolution on beamline I03 at the Diamond Light Source (DLS), Harwell. The crystal belongs to space group P2_1_ with unit cell dimensions *a* = 77.3 Å, *b* = 34.7 Å, *c* = 84.7 Å and *α* = *γ* = 90°, *β* = 102.1°. SeMet crystals were grown in hanging-drops containing a 2:1 ratio of concentrated protein solution : reservoir solution. SeMet-substituted crystals suitable for data collection were obtained using a protein concentration of 1.9 mg ml^−1^ and a reservoir solution of 100 mM MMT pH 6.0 (dl-malic acid, MES and Tris buffers in a molar ratio of 1:2:2), 20% (w/v) PEG 3350, 2% (v/v) DMF. Crystals were soaked in a cryoprotectant solution consisting of the reservoir solution containing 20% (v/v) glycerol before being cryocooled in liquid nitrogen. X-ray diffraction data were collected to 2.1 Å resolution on beamline I24 at DLS. The crystal belongs to space group P2_1_ with cell dimensions *a* = 51.4 Å, *b* = 35.6 Å, *c* = 63.3 Å and *α* = *γ* = 90°, *β* = 92.8°.

Diffraction datasets obtained from the SeMet derivative and native crystals were processed using the automated data processing pipeline Xia2 (Winter, 2009) with options that run XDS (Kabsch, 2010). Data were merged using AIMLESS (Evans, 2006). The structure of SirA–DnaA^DI^ was solved by single-wavelength anomalous dispersion (SAD) phasing. Heavy atom substructure determination, density modification and model building were carried out using the CRANK (Pannu *et al.*, 2011) pipeline available within the Collaborative Computational Project No. 4 (CCP4) program suite (Winn *et al.*, 2001). Nine selenium atom sites were identified using SHELX C/D (Sheldrick, 2008) and their positions refined using BP3. The correct hand for the phases was identified using SOLOMON (Abrahams and Leslie, 1996) and density modification carried out in PARROT (Cowtan, 2010) before atomic model building in BUCCANEER (Cowtan, 2006). The SeMet–SirA–DnaA^DI^ model was partially refined using maximum-likelihood methods in REFMAC (Murshudov *et al.*, 1997) and manual model building in COOT (Emsley and Cowtan, 2004). The partially refined SeMet–SirA–DnaA^DI^ model was used as a model for the solution of native SirA–DnaA^DI^ by molecular replacement with MOLREP (Vagin and Teplyakov, 1997), the search for SirA molecules preceding that for DnaA^DI^ domains. The native SirA–DnaA^DI^ model was refined through iterative cycles of refinement in REFMAC and manual model building in COOT to an *R*-factor of 13.1 (*R*_free_ = 19.7). Refinement statistics are shown in Table 1.

The atomic co-ordinates and crystallographic structure factors have been deposited in the Protein Data Bank with the Accession Code 4TPS.

### SEC-MALLS

SEC-MALLS analysis of DnaA^DI^ and SirA–DnaA^DI^ was carried out at a range of protein concentrations: DnaA^DI^ was analysed at 1.0 mg ml^−1^, 2.5 mg ml^−1^ and 5.0 mg ml^−1^ and SirA–DnaA^DI^ was analysed at 0.5 mg ml^−1^, 1.0 mg ml^−1^ and 2.5 mg ml^−1^. For each run, 100 μl of sample was loaded onto a Superdex 75 HR 10/30 size-exclusion column equilibrated with 50 mM Tris pH 8.5, 200 mM KCl at a flow rate of 0.5 ml min^−1^. The eluate was analysed successively by a SPD20A UV/Vis detector, a Wyatt Dawn HELEOS-II 18-angle light scattering detector and a Wyatt Optilab rEX refractive index monitor as described previously (Colledge *et al.*, 2011). Data were analysed with Astra software (Wyatt).

### Solubility assay

*E. coli* BL21 (DE3), harbouring wild type and mutated pET-YSBLIC3C-DnaA^DI^SirA plasmids were grown in 200 ml LB-kanamycin until the A_600_ reached ∼ 0.6. A portion of cells (uninduced) was set aside and grown separately while IPTG was added to the remaining cells. After a further 4 h growth, aliquots of the uninduced (U) and induced (I) cells were taken and used to prepare total cell samples. The remaining cells from the induced culture were harvested by centrifugation and the cell pellets were re-suspended in 20 ml of 50 mM Tris pH 8.5, 200 mM KCl, 20 mM imidazole, 10 mM BME. Cells were lysed by sonication and the lysate clarified by centrifugation. An aliquot of this soluble fraction (S) was retained. The remaining lysate was loaded onto a 1 ml HisTrap FF crude Ni-affinity column (GE Healthcare), washed with 6 ml re-suspension buffer, and bound protein eluted with 4 ml of 50 mM Tris pH 8.5, 200 ml KCl, 500 mM imidazole, 10 mM BME and the eluate (E) was collected. For each of the wild type and alanine variants, samples of the total fractions from uninduced (U) and induced (I) cells together with the soluble lysis (S) and high imidazole column eluate (E) fractions were analysed by SDS-PAGE followed by Coomassie blue staining.

### *B. subtilis* strains, media and growth conditions

Strains used in this study are listed in Table S3. Nutrient agar (Oxoid) was used as the solid media for growth of *B. subtilis*. LB medium was used for growing cells to extract genomic DNA. Antibiotics were added to the growth media as required: chloramphenicol (5 μg ml^−1^), spectinomycin (50 μg ml^−1^). To induce sporulation *Bacillus subtilis* cells grown in hydrolysed casein media at 37°C were induced to sporulate according to the resuspension method of Sterlini and Mandelstam (1969) as modified by Partridge and Errington (1993).

### Microscopy

After induction of sporulation, samples were taken every 30 min and visualized using fluorescence microscopy. Microscopy was performed using glass slides covered with a ∼ 1.5% agarose pad containing sporulation media. A glass coverslip (0.17 mm VWR) covered cells immobilized on the agarose pad. The dye FM5-95 was added to the agar pads to visualize the membrane (2.9 μg ml^−1^ final). To visualize the nucleoid the DNA was stained with 4′-6-diamidino-2-phenylindole (DAPI 2.5 μg ml^−1^ final). Microscopy was performed on an inverted epifluorescence microscope (Zeiss Axiovert 200M) fitted with a Plan-Neofluar objective (Zeiss 100×/1.30 Oil Ph3). Light was transmitted from a 300 Watt xenon arc-lamp through a liquid light guide (Sutter Instruments) and images were collected using a Sony CoolSnap HQ cooled CCD camera (Roper Scientific). All filters were Modified Magnetron ET Sets from Chroma and details are available upon request. Digital images were acquired and analysed using METAMORPH software (version V.6.2r6).

### Western blot analysis

Proteins were separated by electrophoresis using a NuPAGE 4–12% Bis-Tris gradient gel run in MES buffer (Life Technologies) and transferred to a Hybond-P PVDF membrane (GE Healthcare) using a semi-dry apparatus (Hoefer Scientific Instruments). Proteins of interest were probed with polyclonal primary antibodies and then detected with an anti-rabbit horseradish peroxidase-linked secondary antibody using an ImageQuant LAS 4000 mini digital imaging system (GE Healthcare).
