# Supplementary figures and images for: Structure and interactions of the Bacillus subtilis sporulation inhibitor of DNA replication, SirA, with domain I of DnaA
Source: Mol Microbiol. 2014 Aug 5;93(5):975–91. doi: 10.1111/mmi.12713 (PMC4285326; doi:10.1111/mmi.12713)

# Supplementary Figure 1

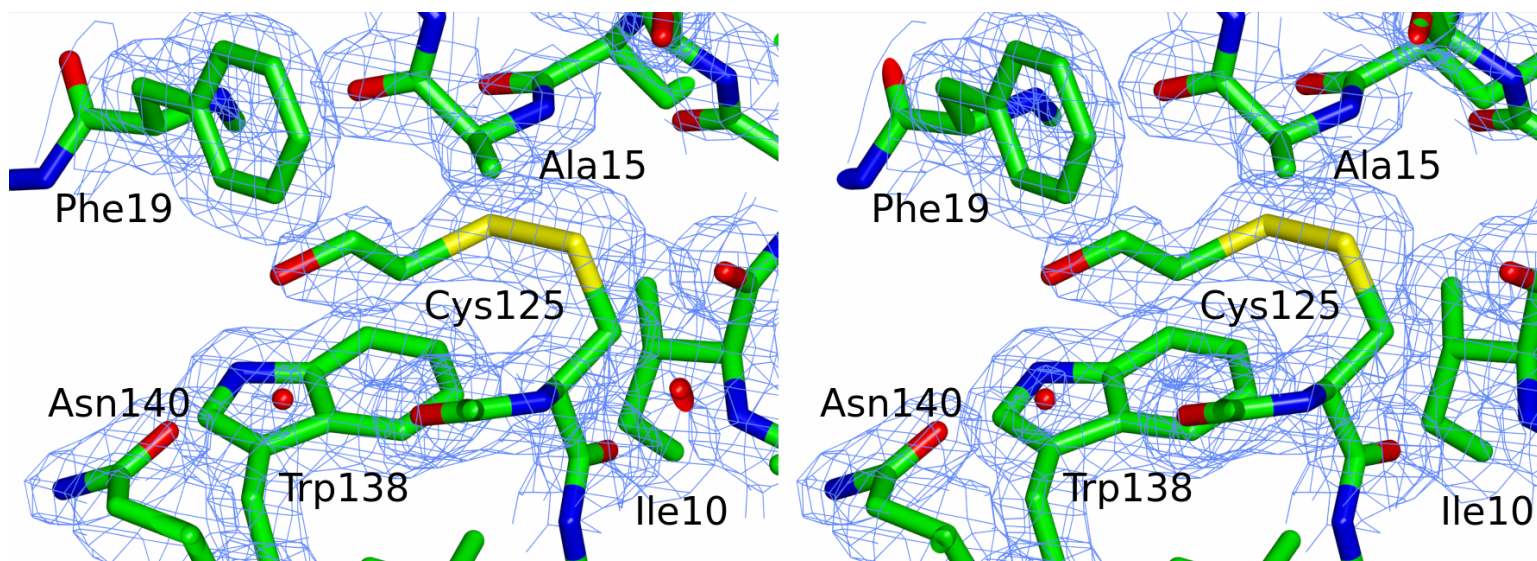

Supplement: Supplementary file 1 — Supporting information [file mmi0093-0975-SD1.pdf]

# Supplementary Figure 2

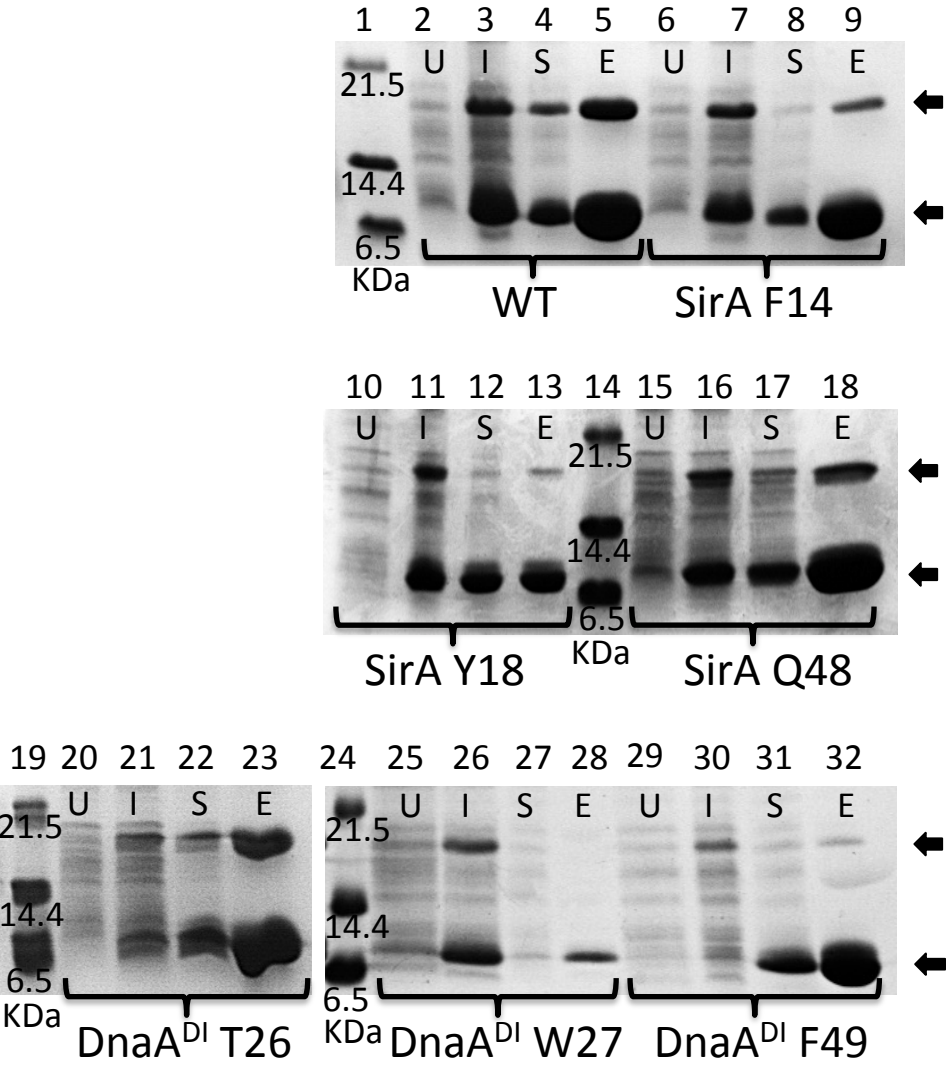

Supplement: Supplementary file 2 — Supporting information [file mmi0093-0975-SD2.pdf]
