# Supplementary material for: Structure and interactions of the Bacillus subtilis sporulation inhibitor of DNA replication, SirA, with domain I of DnaA
Source: Mol Microbiol. 2014 Aug 5;93(5):975–91. doi: 10.1111/mmi.12713 (PMC4285326; doi:10.1111/mmi.12713)
Supplement: Supplementary file 3 — Supporting information [file mmi0093-0975-SD3.pdf]

Supplementary Experimental Procedures

Strains

Standard techniques were used for strain construction (Harwood, C. R., Cutting, 1990). Transformation of competent *B. subtilis* cells was performed using an optimized two-step starvation procedure as previously described (Anagnostopoulos, C., Spitzen, 1961; Hamoen, Smits, de Jong, Holsappel, & Kuipers, 2002). General manipulation of DNA was performed as described (Sambrook, J., Russell, 1989). All plasmids were verified by sequencing.

sirA-gfp *fusions and* sirA *mutants*

In order to place derivatives of *sirA* into the *B. subtilis* chromosome at its endogenous locus a specialized vector was designed that allowed integration by double cross-over. In brief, *sirA* was fused to 3' end of the *gfp* in pSG1729 (linker: PYKKMSRLRIHGPPLE) so that the fusion replaced the 5' fragment of *amyE* (Lewis & Marston, 1999). The xylose inducible promoter was replaced by the region upstream of *sirA* containing both the promoter and ribosome binding site. Finally, a 3' fragment of *tkt* was integrated upstream of the spectinomycin cassette (replacing the 3' fragment of *amyE*) to allow for selection of double cross-over recombination events (pHM349). *sirA* mutants were amplified from expression vectors using PCR and cloned into pHM349 replacing the wild-type gene.

***Overexpression of* SirA proteins**

To overexpress wild-type and mutant SirA in *B. subtilis, sirA* genes were integrated into the *amyE* locus by double-crossover and placed under the control of an IPTG promoter using pDR111 exactly as previously described (Wagner *et al.* 2009). Genomic DNA from strains NR3(*gfp-sirA*); NR130(*gfp-sirA*^{F14A}); NR156(*gfp-sirA*^{Y18A}) and NR131(*gfp-sirA*^{Q48A}) was used as templates for PCR.

dnaA *Mutants*

dnaA mutants were amplified from expression vectors using PCR and cloned into pHM327 (Scholefield, Errington, & Murray, 2012) replacing the wild-type gene.

Supplementary References

Anagnostopoulos, C., Spitzen, J. (1961). Requirements for Transformation in *Bacillus subtilis*. *Journal of Bacteriology*, *81*, 741–746.

Hamoen, L. W., Smits, W. K., de Jong, A., Holsappel, S., & Kuipers, O. P. (2002). Improving the predictive value of the competence transcription factor (ComK) binding site in *Bacillus subtilis* using a genomic approach. *Nucleic Acids Research*, *30*(24), 5517–28.

Harwood, C. R., Cutting, S. M. (1990). *Molecular Biological Methods for Bacillus*. Wiley.

Lewis, P. J., & Marston, A. L. (1999). GFP vectors for controlled expression and dual labelling of protein fusions in Bacillus subtilis. *Gene*, *227*(1), 101–10.

Sambrook, J., Russell, D. W. (1989). *Molecular Cloning: A Laboratory Manual*. Cold Spring Harbor Laboratory Press.

Scholefield, G., Errington, J., & Murray, H. (2012). Soj/ParA stalls DNA replication by inhibiting helix formation of the initiator protein DnaA. *The EMBO Journal*, *31*, 1542–55.

Supplementary Tables

Table S1: Primers for the construction of pET-YSBLIC3C-DnaA^{DI}SirA

Construct	Primer	
pET-YSBLIC3C-DnaA^{DI} SirA	DnaA^{DI} Forward	5'–CCAGGGACCAGCAATGGAAAATATATTAG ACCTGTGGAACCAAGCC–3'
pET-YSBLIC3C-DnaA^{DI} SirA	DnaA^{DI} Reverse	5'–CGCGGGCGGCCGTTATTGAGGAATGACAA ACTTAATGCTC–3'
pET-YSBLIC3C-DnaA^{DI} SirA	SirA Forward	5'–GCGGGCCCGGCCATGGAACGTCACTACTAT ACGTACCTGATC–3'
pET-YSBLIC3C-DnaA^{DI} SirA	SirA Reverse	5'–GAGGAGAAGGCGTTAGACAAAATTTCTTTC TTTCACCGGATTT–3'

Table S2: Oligonucleotides used for the site-directed mutagenesis of pET-YSBLIC3C-DnaA^{DI}SirA

pET-YSBLIC3C-DnaA^{DI}T26A SirA	5'– CCGAGTTTTGAGGCTTGGATGAAGTCAACC –3'
	5'– GGTTGACTTCATCCAAGCCTCAAAACTCGG –3'
pET-YSBLIC3C-DnaA^{DI}W27A SirA	5'– CGAGTTTTGAGACTGCGATGAAGTCAACC –3'
	5'– GGTTGACTTCATCGCAGTCTCAAAACTCG –3'
pET-YSBLIC3C-DnaA^{DI}F49A SirA	5'– CGGCTCCCAATGAAGCTGCCAGAGACTGGC – 3'
	5' – GCCAGTCTCTGGGCAGCTTCATTGGGAGCCG – 3'
pET-YSBLIC3C-DnaA^{DI}SirAF14A	5' – CCTGATCAAAGAGGAAGCTGCCAATCACTATTTCGG – 3'
	5' – CCGAAATAGTGATTGGCAGCTTCCTCTTTGATCAGG – 3'
pET-YSBLIC3C-DnaA^{DI}SirAY18A	5' – GGAATTTGCCAATCACGCTTTCGGCCGGGAATCGG – 3'
	5' – CCGATTCCCGGCCGAAAGCGTGATTGGCAAATTCC – 3'
pET-YSBLIC3C-DnaA^{DI}SirAQ48A	5' – GAAATGACAGAGAAAGCGATTCAATATATTACAC – 3'
	5' – GTGTAATATATTGAATCGCTTTCTCTGTCATTTC – 3'

Table S3: ***B. subtilis*** **Strain list**

Strains	Genotype	Reference
168ed	*trpC2*	Kobayashi et al., 2003
NR3	*trpC2 gfp-sirA*::*spec*	This work
NR5	*trpC2 gfp-sirA*::*spec dnaA*^{A50V}::*cat*	This work
NR130	*trpC2 gfp-sirA*^{F14A}::*spec*	This work
NR131	*trpC2 gfp-sirA*^{Q48A}::*spec*	This work
NR154	*trpC2 gfp-sirA*::*spec dnaA*^{N47H}::*cat*	This work
NR156	*trpC2 gfp-sirA*^{Y18A}::*spec*	This work
NR164	*trpC2 gfp-sirA*::*spec aprE::cat*(P_{xyl}-*tetR-mCherry) spo0J*::(*tetO*$_{150}$ *neo*)	This work
NR168	*trpC2 gfp-sirA*::*spec dnaN'*Ω(*dnaN-mCherry spec*)	This work
NR171	*trpC2 amyE*::*spec(*$P_{hyperspank}$-*sirA)*	This work
NR172	*trpC2 amyE*::*spec(*$P_{hyperspank}$-*sirA*^{F14A}*)*	This work
NR173	*trpC2 amyE*::*spec(*$P_{hyperspank}$-*sirA*^{Q48A}*)*	This work
NR174	*trpC2 amyE*::*spec(*$P_{hyperspank}$-*sirA*^{Y18A}*)*	This work
JWV160	*sacA::neo(*P_{sda}-*gfp) dnaN'*Ω(*dnaN-mCherry spec*)	Veening et al.,2009
HM130	*trpC2 spo0J*::(*tetO*$_{150}$ *neo*) *cgeD*::($P_{pen(mutTTAG)}$-*tetR-yfp tet*)	Murray et al.,2008

Supplementary Figure Legend

Figure S1: 2-Mercaptoenthanol Adduct of SirA: Stereoview of residues in the neighbourhood of Cys^{125} of SirA, showing 2-mercaptoenthanol connected to the thiol of the side chain through a disulphide bond. Electron density is shown in blue contoured at 1σ. The model is coloured by atom with carbon in green, oxygen in red, nitrogen in blue and sulphur in yellow.

Figure S2. The SirA-DnaA^{DI} interface analysed by site-directed mutagenesis. SDS-polyacrylamide gel electrophoresis. Cultures of cells harbouring plasmids encoding wild type and alanine-substituted variants of His-tagged DnaA^{DI} and SirA were grown and total cell fractions were prepared from uninduced (U) and induced (I) cell pellets. Soluble cell lysates were prepared (S) and loaded onto a Ni-NTA column. High imidazole eluate (E) fractions were collected for analysis. Lanes 1, 14, 19 and 24 contain molecular weight markers. Uninduced (U), Induced (I), Soluble (S) and Eluate (E) fractions were loaded for the wild type proteins in Lanes 2-5, and for wild type DnaA^{DI} produced together with SirA mutants as follows; SirA(F14A) in Lanes 6-9:

SirA(Y18A) in Lanes 10-13: SirA(Q48A) in Lanes 15-18. Samples of native SirA and the DnaA^{DI} variants were loaded as follows; DnaA^{DI}(T26A) in Lanes 20-23: DnaA^{DI}(W27A) in Lanes 25-28; DnaA^{DI}(F49A) in Lanes 29-32. The positions of the bands associated with SirA and DnaA^{DI} are indicated by the upper and lower arrows respectively in each gel.
